# NICEFIT—A Prospective, Non-Interventional, and Multicentric Study for the Management of Idiopathic Pulmonary Fibrosis with Antifibrotic Therapy in Taiwan

**DOI:** 10.3390/biomedicines10102362

**Published:** 2022-09-22

**Authors:** Shih-Lung Cheng, Chau-Chyun Sheu, Chih-Feng Chian, Jeng-Yuan Hsu, Kuo-Chin Kao, Liang-Wen Hang, Ching-Hsiung Lin, Wen-Feng Fang, Hao-Chien Wang, Diahn-Warng Perng

**Affiliations:** 1 Department of Chemical Engineering and Materials Science, Yuan Ze University, Taoyuan City 320, Taiwan; 2 Department of Pulmonary, Far Eastern Memorial Hospital, New Taipei City 220, Taiwan; 3 Division of Pulmonary and Critical Care Medicine, Department of Internal Medicine, Kaohsiung Medical University Hospital, Kaohsiung Medical University, Kaohsiung 807, Taiwan; 4 Division of Pulmonary and Critical Care Medicine, Department of Internal Medicine, Tri-Service General Hospital, National Defense Medical Center, Taipei 114, Taiwan; 5 Division of Clinical Research, Taichung Veterans General Hospital, Taichung 407, Taiwan; 6 Department of Thoracic Medicine, Linkou Chang Gung Memorial Hospital, Taoyuan 333, Taiwan; 7 Department of Pulmonary, China Medical University Hospital, Taichung 404, Taiwan; 8 Institute of Genomics and Bioinformatics, National Chung Hsing University, Taichung 402, Taiwan; 9 Ph.D. Program in Translational Medicine, National Chung Hsing University, Taichung 402, Taiwan; 10 Department of Recreation and Holistic Wellness, MingDao University, Changhua 403, Taiwan; 11 Division of Chest Medicine, Department of Internal Medicine, Changhua Christian Hospital, Changhua 403, Taiwan; 12 Department of Internal Medicine, Division of Pulmonary and Critical Care Medicine, Kaohsiung Chang Gung Memorial Hospital, Chang Gung University College of Medicine, Kaohsiung 833, Taiwan; 13 Department of Medicine, National Taiwan University Cancer Center, Taipei 100, Taiwan; 14 Department of Chest Medicine, School of Medicine, National Yang-Ming Chiao-Tung University, Taipei Veterans General Hospital, Taipei 112, Taiwan

**Keywords:** nintedanib, Taiwan, idiopathic pulmonary fibrosis, lung function, real-world registry, pirfenidone

## Abstract

Idiopathic pulmonary fibrosis (IPF) causes progressive lung fibrosis with subsequent fatality and has limited treatment options. NICEFIT is the first Taiwan-based prospective, observational, and non-interventional registry for IPF progression under routine clinical practice in Taiwan. Data on 101 patients (aged 74.6 ± 9.1 years and 83.2% men) with IPF were collected over 2 years (2018−2020) from medical centers in Taiwan at baseline, 1 month, and subsequent 3-month intervals. Treated patients (n = 88) received the antifibrotics nintedanib or pirfenidone, compared with the untreated group (n = 13). The 2-year assessment revealed overall preserved lung functionality in the treated patients, with insignificant changes from baseline for percent predicted forced vital capacity or FVC (±1.7%). The presence of respiratory comorbidities significantly increased the risk of both AE and death (with or without AE) over the full study duration. Furthermore, the decline of predicted FVC significantly increased with the risk of acute exacerbations (AE) in the second year. Overall, antifibrotic medication was beneficial in stalling IPF progression, reducing AEs, and delaying mortality in the treated cohort, despite their lower baseline lung functions. Further, no new safety concerns over antifibrotic treatments were observed for the Taiwanese population.

## 1. Introduction

Idiopathic pulmonary fibrosis (IPF) is a chronic lung disease characterized by progressive dyspnea and decline in pulmonary function [1]. Decreased forced vital capacity (FVC) is an important clinical indication [2,3]. Fatality from IPF occurs due to respiratory failures with acute exacerbations constituting nearly 10–20% these cases [1]. Globally, the incidence rate of IPF varies, with Europe and North America having the highest incidence (3 to 9 cases per 100,000 population per year) followed by Asia and South America (0.4 to 3.8 cases per 100,000 population per year) [4]. An insurance claims-based study of IPF patients in Taiwan has reported an incidence rate of 0.6–1.4 per 100,000 population per year and an IPF prevalence rate of 0.7–6.4 cases per 100,000 population per year, with both rates showing an annual increase between 1997–2007 [5]. Whereas causative factors are unknown, predisposing risk factors for IPF include infectious agents, gastroesophageal reflux, and genetic mutations that compromise airway immunity [1]. Furthermore, older (>50 years) male adults with a smoking habit are a high-risk group [6].

During IPF, the alveolar epithelium undergoes remodeling to fibrotic tissue as a consequence of constant inflammation and mis-repair [7]. Dyspnea related to IPF is often misdiagnosed as chronic obstructive pulmonary disease (COPD) or heart ailments, delaying clinical intervention and shortening median survival [8]. A retrospective study in a North American population indicated a median survival of 2.8 to 3 years, with 20–30% surviving up to 5 years post diagnosis [9]. In Taiwan, the median survival post diagnosis is much lower, at 0.9 years [5]. To overcome diagnostic challenges, the joint statement issued by the American Thoracic Society, the European Respiratory Society, the Japanese Respiratory Society, and the Latin American Thoracic Association 2011, specified the presence of usual interstitial pattern (UIP) in high-resolution computerized tomography (HRCT) as the diagnostic guideline for IPF [2].

Although there is no treatment, the progression of IPF can be slowed by early intervention with antifibrotic agents [10]. Nintedanib is an intracellular tyrosine kinase inhibitor that stalls vascular endothelial growth factor (VEGF)- and platelet-derived growth factor (PDGF)-based cellular signals, which are driven by transforming growth factor-β (TGF-β) for tissue remodeling in IPF [11,12] and has shown significant reduction in the annual rate of FVC decline (over placebo) in randomized control trials (INPULSIS-1 and INPULSIS-2) [13]. Pirfenidone, an antifibrotic agent that blocks profibrogenic mediators such as TGF-β and TNF-α [14], was evaluated in the ASCEND clinical trial where it demonstrated decreased IPF progression (over placebo), with tolerable side effects [15]. Both agents are currently approved for IPF in Europe and the United States of America [10].

Medical practitioners in Taiwan follow the conditional prescription of nintedanib or pirfenidone for patients with IPF, in accordance with the international consensus statement of 2015 [16]. Nintedanib (OFEV^®^) and pirfenidone (Pirespa^®^) have been available in Taiwan since late 2015 and 2016, respectively, with both antifibrotic drugs being recommended for IPF management in 2018 [1]. The National Health Insurance (NHI) in Taiwan does not reimburse antifibrotic therapy for IPF in patients with FVC exceeding 80%. This is reflected in the sampling of the treated and untreated cohorts in this study, wherein all treated patients have lower pulmonary functionality than untreated individuals at baseline. However, in the absence of randomized control trials or registries describing IPF progression in Taiwan, NICEFIT is the first prospective, long-term, non-interventional study of patients with a recent IPF diagnosis who are availing routine management under NHI. The objective of the study was to observe the epidemiology of IPF in the context of its current management procedure in Taiwan.

## 2. Materials and Methods

### 2.1. Study Design

This prospective, non-interventional, multicentric, Taiwan-based study enrolled 101 patients across 10 clinical centers in Taiwan (NCT03242759) from 31 August 2017 to 27 February 2018. Only clinics which had access to all treatment options approved for IPF in Taiwan were chosen for data collection to eliminate selection bias at the site of treatment. To negate selection bias at the patient level, study enrollment took place in a consecutive manner across clinics. IPF progression was followed over 100 weeks. On the basis of treatment with the antifibrotics nintedanib and pirfenidone, patients were segregated into the treated group (receiving antifibrotic therapy) and the untreated group. Baseline readings for primary and secondary outcomes (see below) were collected on the date of enrollment. This was followed by data collection at 1 month post interval and at 3-month intervals thereafter. No interim analyses were conducted.

### 2.2. Inclusion and Exclusion Criteria

Patients were included if they were ≥20 years of age and received IPF diagnosis within 6 months of study enrollment based on the presence of UIP by HRCT or HRCT and surgical lung biopsy as specified by recent The American Thoracic Society/European Respiratory Society/Japanese Respiratory Society and Latin American Thoracic Association (ATS/ERS/JRS/ALAT) IPF guidelines [2]. Only those patients in a position to provide written informed consent prior to participation and those who could attend follow-up assessments with the participating physician during the study period were included. Patients were excluded if their diagnosis involved other known causes of interstitial lung disease (ILD), such as domestic and occupational environmental exposures, connective tissue disease, or drug toxicity. Furthermore, patients who were expected to receive lung transplantation within the next 6 months or those who were participants in ongoing clinical trials were excluded.

### 2.3. Primary Outcomes

Primary outcomes for assessing lung function profile included annual percentages of decline from baseline in (i) FVC, (ii) diffusion of carbon monoxide in lungs (DL_CO_), (iii) oxygen saturation (SpO_2_), (iv) total lung capacity (TLC), and (v) inspiratory capacity (IC) as measured through spirometry.

### 2.4. Secondary Outcomes

Secondary outcomes included time to first acute exacerbation of IPF after enrollment, mortality (with cause of death), and time to death along with annual change from baseline in (i) St. George’s respiratory questionnaire (SGRQ), (ii) COPD assessment test (CAT), and (iii) 6 min walk test (6MWT).

### 2.5. Antifibrotic Drug Treatment

Patients on nintedanib (OFEV^®^, manufactured by Catalent Germany Eberbach GmbH and primary & secondary packaged by Boehringer Ingelheim Pharma GmbH & Co. KG, Germany) received one of the three dosages: 150 mg BID (88%), 150 mg QD (10.8%), or 150 mg QOD (1.2%) (Table S1). Pirfenidone (Pirespa^®^, manufactured and packaged by Shionogi, Japan & Co., Ltd., Osaka, Japan) was administered as 267 mg TID (i.e., 801 mg/day) in the first week, 534 mg TID (i.e., 1602 mg/day) in the second week, and 801 mg TID (i.e., 2403 mg/day) from the third week of treatment. Dosages above 2403 mg/day were avoided, and a patient did not exceed 3 doses per day. Adverse reactions to OFEV were managed through dose reduction or temporary interruptions in therapy. OFEV was re-initiated once the adverse reactions resolved to acceptable levels. The re-initiation protocol involved resumption of OFEV at the full dosage (150 mg BID). Adverse reactions to pirfenidone were managed through temporary dosage reductions or treatment interruptions until resolution of symptoms (e.g., gastrointestinal events, photosensitivity reaction, or rash). For treatment interruption of less than 14 days, pirfenidone dosages prior to the interruption were resumed. For interruptions of more than 14 days, pirfenidone was re-initiated with the dosage regimen for the first 2 weeks, leading up to the full maintenance dosage (801 mg TID).

Drugs were switched in patients in cases of adverse drug reactions, poor IPF prognosis, or withdrawal of consent.

### 2.6. Data Collection Procedure

Data acquisition was non-interventional and included questionnaires, hospital discharge files, primary clinical records, electronic medical records, clinical databases, administrative records (e.g., eligibility files, prescription drug files), biological measurements, and occupational exposure records collected during the routine clinical practice. For data collection on baseline and follow-up visits, medical history of IPF, and comorbidities, please refer to Supplementary Methods. The procedure for gene polymorphism, as well as for serological and biomarker testing, is also provided in the Supplementary Methods.

#### 2.6.1. IPF-Related Acute Exacerbations

Acute exacerbations were defined as acute, clinically significant deteriorations of an unidentifiable cause in patients with underlying IPF. The date of an event was recorded as acute exacerbations if clinical manifestation included (i) unexplained worsening or development of dyspnea within 30 days of follow-up, (ii) new diffuse pulmonary infiltrates as seen on HRCT and/or chest radiography, or (iii) parenchymal abnormalities without pleural effusions since previous visit. Known causes for pulmonary deterioration such as infection, left heart failure, pulmonary embolism, or any identifiable cause of acute lung injury were excluded.

#### 2.6.2. Six-Minute Walk Test (6MWT)

The 6MWT provides information about functional pulmonary capacity, a response to therapy indicative of the prognosis of IPF. The test measures the distance someone can walk quickly on a flat, hard surface in 6 min. The 6MWT indicates the subject’s ability to perform daily physical activities. Results were collected at weeks 25, 50, and 100. Details of the date of the test, distance covered (m), and SpO_2_ (%) while at rest and during exercise, were recorded.

#### 2.6.3. St. George’s Respiratory Questionnaire (SGRQ)

SGRQ is a self-assessment questionnaire covering 40 items related to the health-related quality of life of patients with airway obstructing pulmonary diseases. SGRQ assesses the patient’s symptoms, limitations to physical activity, and social and emotional well-being. Each item is weighted for distress on a scale of 0 to 100, with a higher score implying poorer health-related quality of life [17]. A change in scale by 4 units is considered to be clinically relevant and requires a clinician’s attention. The dates of visit for the SGRQ assessment along with the respective SGRQ scores were recorded at baseline and with every follow-up visit.

#### 2.6.4. COPD Assessment Test (CAT)

CAT is an 8-item health assessment test for understanding the impact of COPD on quality of life. COPD is an important comorbidity with IPF. The CAT score is measured on a scale of 0 to 40, with a higher score indicating a higher adverse impact of COPD on a patient’s lifestyle [18]. A minimum difference of 2 points in CAT score indicates a clinically relevant change [19]. The dates of visit for the CAT assessment along with the respective CAT scores were recorded at baseline and with every follow-up visit.

#### 2.6.5. Mortality

Fatal events were recorded to reflect the date of death and if the cause of fatality was (i) IPF-related (e.g., respiratory failure, acute exacerbation), (ii) comorbidity-related (e.g., coronary, cerebrovascular, pulmonary embolism or hypertension, lung cancer), (iii) other causes (e.g., atypical pneumonia, myelodysplastic syndrome, acute kidney injury, intraventricular bleeding), or (iv) unknown.

#### 2.6.6. Adverse Events (AE) and Adverse Reactions

An AE was defined as any untoward medical occurrence in a patient receiving antifibrotic medication that did not necessarily have a causal relationship with the medication. Events that were fatal or life threatening and led to prolonged hospitalization or significant disability were scored as serious AEs. An adverse reaction was defined as a response to the antifibrotic medication that was noxious and unintended, implying a causal relationship with the medication. The clinical investigator maintained detailed records of all adverse drug reactions (serious and mild) and fatal adverse events. An AE was considered an adverse drug reaction if the outcome (i) was consistent with the pharmacological profile of the drug, (ii) manifested within reasonable time of drug exposure or was dose dependent in magnitude, (iii) reproduced after discontinuation and re-introduction of the drug, or (iv) if the event was otherwise not known to occur in a population unexposed to the drug.

### 2.7. Statistical Analyses

Statistical analyses were performed using the Statistical Analysis Software (SAS^®^) version 9.4, SAS Institute, Cary, NC, USA. Analysis of primary and secondary outcomes was conducted by calculating the following: mean (for annual change/decline from baseline and annual change over 100 weeks); standard deviation (SD); minimum, lower quartile (Q1); median, upper quartile (Q2); maximum value; and 95% confidence intervals (CI). Difference in baseline parameters within groups was assessed by Wilcoxon signed rank test or t-test, as applicable, with a *p*-value < 0.05. Time to first acute exacerbation and all-cause mortality were analyzed by Kaplan–Meier estimates, including time of incidence and time to event. Kaplan–Meier survival analysis was performed for overall survival at 100 weeks. Time to death was calculated from the date of enrollment to the date of death from any cause. The median, range, and 95% CI for median survival time were reported. To correct for bias from missing values, imputation was permitted in the analysis of primary and secondary outcomes on each case basis, considering the extent and distribution of missing values. Imputation was not performed for missing data on safety analysis. Missing data for time to event endpoints were covered by standard survival analysis techniques.

## 3. Results

### 3.1. Patient Characteristics

**Figure 1:**
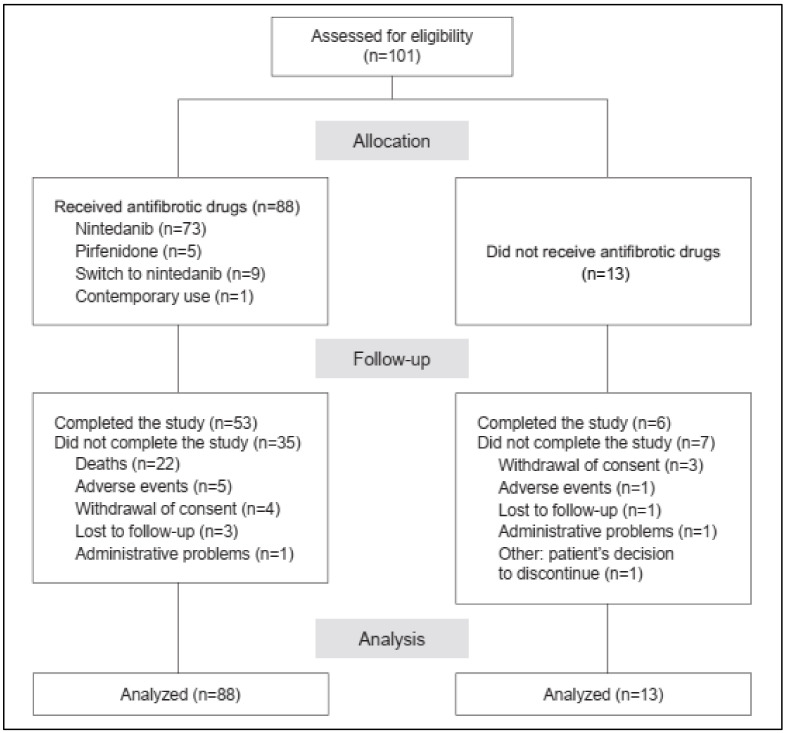
**A summary of patient disposition.** A total of 101 eligible patients were recruited and allocated to the treated group receiving the antifibrotics nintedanib and pirfenidone (n = 88) or the untreated group (n = 13).

Patients were enrolled across 10 clinical centers in Taiwan following the main inclusion criteria of recently diagnosed IPF (within 6 months of the date of enrollment). Diagnosis was based on the IPF 2011 diagnostic guidelines for the presence of UIP in an HRCT scan (Table S2). The main reasons for exclusion were requirement of lung transplant within 6 months of the date of enrollment and patient’s participation in ongoing clinical trials. Of the 101 patients (Figure 1), 87.1% (n = 88), designated as the treated group, received antifibrotic drugs (nintedanib or pirfenidone), whereas 12.9% (n = 13) who did not receive antifibrotic treatment were placed in the untreated group. The majority of the treated group (83%, n = 73) were administered nintedanib and the remainder (5.7%, n = 5) were administered pirfenidone. A few patients receiving nintedanib (n = 9) had switched from pirfenidone during the course of this study due to AEs (60%), poor IPF prognosis (20%), or withdrawal of consent (10%). One patient was on concomitant medication with both drugs. During the 2-year follow-up, 59 patients completed the study. Data on the remaining patients were lost due to consent withdrawal (n = 7), AEs (n = 6), death (n = 22), administrative problems (n = 2), patient’s decision to discontinue (n = 1), and absence at follow-up due to unknown reasons (n = 4) (Figure 1).

There were no significant differences in demographics, IPF diagnostic profile, or comorbidity between the treated and untreated groups (Table 1). Enrolled patients were of East Asian descent, mostly men (83.2%, n = 84), with age (mean ± SD) of 74.6 ± 9.1 years, height (mean ± SD) of 162.7 ± 7.3 cm, and weight (mean ± SD) of 65.3 ± 12.5 kg; hence, BMI (mean ± SD) was 24.6 ± 4.4. Definite UIP in HRCT pattern was identified in 72 patients, with possible UIP in 23 patients and an inconsistent UIP pattern in 3 patients (Table S2). The most frequently encountered comorbidities in the study patients were type 1 or type 2 diabetes mellitus (21.8%), COPD (34.7%), obstructive sleep apnea (16.8%), radiologic emphysema (19.8%), arterial hypertension (47.5%), coronary artery disease (18.8%), gastroesophageal reflux disease (25.7%), and gastric ulcer (11.9%) (Table 1). IPF patients received respiratory-related (65.3%), hypertension-related (20.8%), or cerebral-artery-occlusion-related (17.8%) comorbidity medications (Table S3).

Primary outcomes for pulmonary fitness are described as % annual changes from baseline for FVC, DL_CO_, SpO_2_, TLC, and IC. Baseline characteristics (mean ± SD) for the primary outcome parameters were 73.3 ± 16.6% for FVC (% pred), 45.2 ± 20.1% for DL_CO_ (% pred), 95.8 ± 2.3% for SpO_2_ (%), 77.2 ± 15.5% for TLC (% pred), and 65.6 ± 17.9% for IC (% pred) (Table 2). The baseline parameters (overall mean ± SD) for the secondary outcomes were 33.4 ± 19.8 score for SGRQ assessed health-related quality of life and 11.3 ± 7.1 for CAT assessed quality of life. The mean (% change from baseline) distance, resting SpO_2_, and exercise SpO_2_ for the 6MWT were 329.8 ± 96.8 m, 94.7 ± 3.4%, and 90.9 ± 5.7%, respectively (Table 2).

The percent predicted (mean ± SD) values for pulmonary function between the treated and untreated groups showed significant differences (*p* < 0.05), as patients receiving antifibrotic treatment had poorer baseline readings for lung function, condition of airway obstruction, and quality of life (Table 2).

### 3.2. IPF Progression in the Treated Group

#### 3.2.1. Primary Outcomes

**Figure 2:**
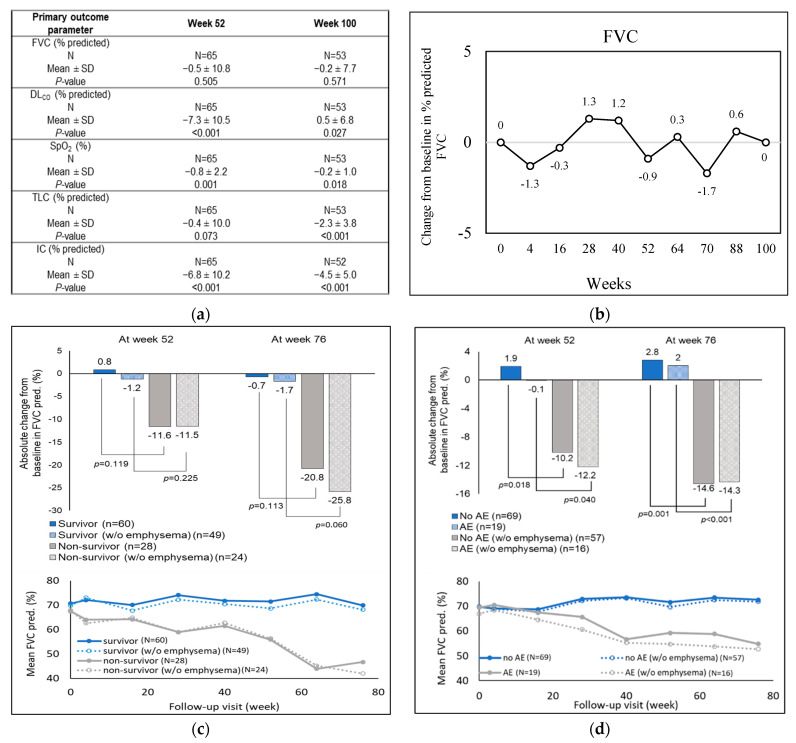
Changes in baseline of primary outcome parameters across the study period in the treated group. (**a**) Annual changes from baseline for the primary lung function parameters, (i) FVC, (ii) DL_CO_, (iii) SpO_2_, (iv) TLC, and (v) IC, as measured through spirometry. (**b**) Changes in percent predicted FVC from baseline. (**c**) Predicted mean and absolute FVC changes in survivors and non-survivors, treated with antifibrotic drugs, and (**d**) predicted mean and absolute FVC changes in treated patients experiencing versus not experiencing at least one acute exacerbation, stratified by emphysema status. AE, acute exacerbation; DL_CO_, diffusion of carbon monoxide in lungs; FVC, forced vital capacity; IC, inspiratory capacity; SpO_2_, oxygen saturation; pred, predicted; TLC, total lung capacity.

Patients treated with nintedanib or pirfenidone showed reduced decline of lung function parameters, from baseline, over 100 weeks of observation (Figure 2a). The mean ± SD of absolute annual change from baseline in FVC was −114.3 ± 441.5 mL at week 52 and −142.5 ± 610.8 mL at week 100 (Figure S1). The change from baseline in percent predicted FVC was insignificant (±1.7%) across the study period, indicating stabilized lung function in treated patients (Figure 2b).

The trends in changes from baseline for absolute and percent predicted values in primary outcomes, across the 2-year study period, are shown in Figure S1. There was no significant change from baseline (mean ± SD) for absolute DL_CO_, which ranged from −2.6 to 0.1 mL/min/mmHg (Figure S1) during the 2-year follow-up. Percent predicted values (mean ± SD) for SpO_2_, TLC, and IC remained close to the baseline for both week 52 (−0.8 ± 2.2, −0.4 ± 10.0, and −6.8 ± 10.2, respectively) and week 100 (−0.2 ± 1.0, −2.3 ± 3.8, and −4.5 ± 5.0, respectively) (Figure 2a). Thus, there were no clinically significant variations in annual changes from baseline in primary endpoints during the entire study period, implying that antifibrotic treatment limited deterioration of pulmonary capacity in patients with IPF.

#### 3.2.2. Secondary Outcomes

Interestingly, survival data revealed that non-survivors had higher absolute and mean reductions in predicted FVC than survivors at weeks 52 and 76 of the study period (Figure 2c). This trend was evident regardless of the emphysema status but did not rise to statistical significance in any comparison (*p* > 0.05). Mean predicted FVC continued to diverge across the duration of the study (Figure 2c). Acute exacerbations were observed in 21.6% (n = 19) of the treated patients after a median follow-up period of 497 days post enrollment. As shown in Figure 2d, acute exacerbation was significantly associated with reductions in absolute predicted FVC at week 52 and week 76 in patients with emphysema (absolute change at week 52: 1.9% vs. −10.2%, *p* = 0.018 and week 76: 2.8% vs. −14.6%, *p* = 0.001), as well as in those without emphysema (week 52: −0.1% vs. −12.2%, *p* = 0.040 and week 76: 2.0% vs. −14.3%, *p* < 0.001).

Mean predicted FVC diverged over the course of the study period. Twenty-eight (31.8%) fatal events were recorded with a median follow-up period of 686 days (Figure S2). Eleven patients died from acute IPF exacerbations, seven patients died from comorbidities, and ten patients died due to non-IPF-related conditions (Figure S2). The treated group showed a median survival period of 708 days (Table S4). The presence of any respiratory comorbidity was observed to significantly increase all-cause mortality (OR 16.286; 95% CI 2.0, 132.9; *p* = 0.009) and risk of AE and death (OR 4.848; 95% CI 1.15, 20.5; *p* = 0.032) over the full study duration (Table 3 and Table 4). Further, the risk of acute exacerbation (but not death) significantly increased with decline in percent predicted FVC between weeks 53–104 (OR 10.887; 95% CI 1.03, 114.8; *p* = 0.047, Table 5).

**Figure 3:**
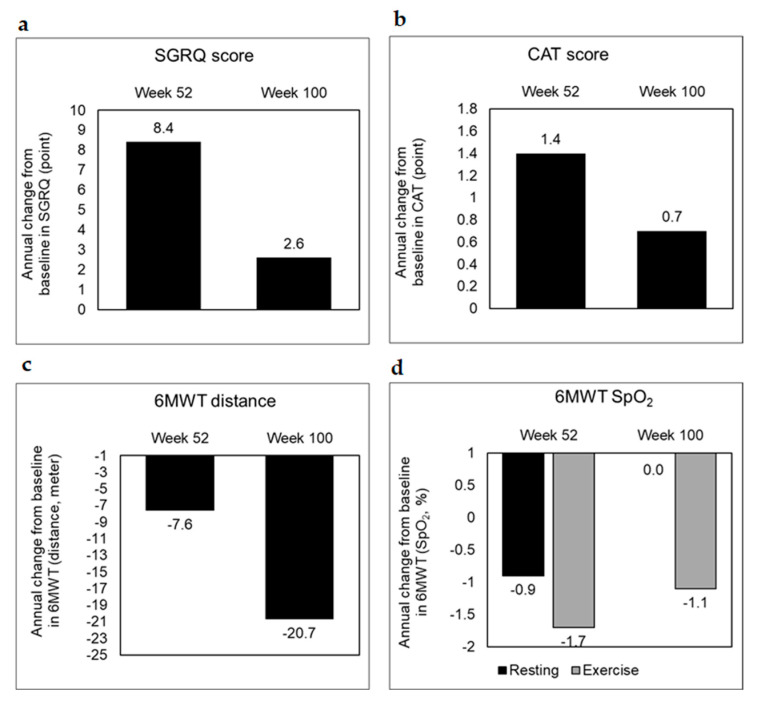
Secondary outcome trends in the treated group. Secondary outcomes with respect to (**a**) SGRQ, (**b**) CAT, and (**c**,**d**) 6MWT were scored as annual changes from the baseline at the end of weeks 52 and 100 to assess health-related quality of life, airway obstruction and exercise-related pulmonary function, respectively. SGRQ, St. George’s respiratory questionnaire; CAT, chronic obstructive pulmonary disease assessment test; 6MWT, 6 min walk test.

Health-related quality of life assessment through SGRQ showed poor prognosis in treated patients with annual changes from baseline for SGRQ worsening to 8.4 ± 16.5 points (95% CI 4.0, 12.8) at week 52 (Figure 3a, Table 6). However, this was stabilized at 2.6 ± 13.4 points (95% CI −1.1, 6.3) by week 100 of ongoing antifibrotic treatment. COPD-like airway obstruction was not exacerbated during the 2-year follow-up period, with annual changes in CAT scores fluctuating less than 2 points (1.4 ± 7.8 points, 95% CI −0.7, 3.5 at week 52 and 0.7 ± 4.4 points, 95% CI −0.5, 1.9 at week 100) (Figure 3b, Table 6). Changes in the 6MWT (m) were recorded at −7.6 ± 60.4 (95% CI −33.1, 17.9) for week 52 and −20.7 ± 36.6 (95% CI −40.9, −0.4) for week 100, indicating a decline in physical fitness of treated subjects towards the end of 2 years (Figure 3c–d, Table 6). Among the 88 patients treated with antifibrotic agents, 64 patients (72.7%) experienced AEs. Common AEs occurring in treated patients included diarrhea (33.0%), followed by idiopathic pulmonary fibrosis (10.2%) and hepatobiliary disorders (5.7%) (Table S5).

### 3.3. IPF Progression in the Untreated Group

The untreated group had fluctuating trends owing to the small size of the cohort (n = 13 or 12.9% of overall study enrolment) allowing for limited interpretation of data.

#### 3.3.1. Primary Outcomes

For primary outcome of lung function parameters, no significant changes from baseline were observed in the untreated group. Changes in percent predicted FVC (mean ± SD) at weeks 52 and 100 were 4.1 ± 7.7 and −2.5 ± 4.5, respectively. A fluctuating trend in mean FVC was observed across the study period. Compared with the mean FVC of 2886.9 mL at baseline, the estimated mean change in FVC was 52.0 mL at week 52 and −210 mL at week 100. Generally, a decline in annual DL_CO_ (% predicted) was noted, with the annual changes in baseline at week 52 and week 100 being −2.8 ± 8.27 and −2.6 ± 6.3, respectively (Table S6). A stable pattern of mean SpO_2_ was observed with annual percent predicted changes from baseline (mean ± SD), being −0.8 ± 0.9 at week 52 and −0.6 ± 0.8 at week 100 (Figure S4), with minor changes of −1.5 to −0.3% over 2 years. Change (mean ± SD) in percent predicted TLC at week 52 was −1.4 ± 12.2 and at week 100 was −3.4 ± 6.9 (Table S6), and reflected a declining trend throughout the study period. The mean changes in TLC were −240.0 mL at week 52 and −340.0 mL at week 100. The change in percent predicted IC (mean ± SD) was −6.1 ± 1.7% at week 52, which was maintained at −6.0 ± 2.4% at week 100 (Table S6). The mean IC ranged from 1305.0 to 1885.0 mL.

#### 3.3.2. Secondary Outcomes

Health-related quality of life assessment through SGRQ (points) revealed annual changes from baseline of 0.2 ± 8.1 (95% CI −6.6, 7.0) for week 52 and 1.6 ± 6.2 (95% CI −4.8, 8.1) for week 100 (Figure S3a, Table S7). Annual changes in CAT scores for airway obstruction were 0.2 ± 2.0 (95% CI −1.4, 1.8) at week 52 and 0.7 ± 2.1 (95% CI −1.5, 2.9) for week 100 (Figure S3b, Table S7). Annual changes in 6MWT were 7.1 ± 31.5 (95% CI −71.2, 85.5) for week 52 and −2. 3± 20.5 (95% CI −186.6, 181.9) for week 100 (Figure S3c,d, Table S7).

Two patients experienced acute exacerbation after a median follow-up period of 521.5 days from study enrollment, of which one patient succumbed to coronary disease at 641 days of follow-up (Table S8). Furthermore, five AEs were recorded across four patients (30.8%, Table S9), with severe AEs presenting in three of these patients. Two serious AEs were noted. (Table S9).

## 4. Discussion

NICEFIT is the first Taiwan-based IPF registry reporting the management of IPF under routine clinical practice in Taiwan, focusing on outcomes of lung function and quality of life in IPF patients. Patients with FVCs of less than 80% are placed on prompt antifibrotic medication facilitated by the reimbursement scheme of NHI. This led to differences in the sampling size of the treated (n = 88) and untreated (n = 13) cohorts in the present study, with the treated cohort presenting poorer lung function parameters (*p* < 0.05) at baseline than the untreated cohort. Despite significantly compromised baseline functionality, antifibrotic therapy with nintedanib or pirfenidone limited further deteriorations of respiratory functions, especially with respect to annual changes from baseline in percent predicted FVC (±1.7%), without adversely affecting the quality of life. Stabilization of FVC with antifibrotic therapy achieved significant delays in acute exacerbations while maintaining survival (median survival = 708 days) in the treated group, both of which are the core aims of IPF treatment. An FVC decline was found to be associated with increased risk of AE irrespective of concomitant emphysema (*p* = 0.001, week 76) in the present study. Additionally, decline in percent predicted FVC significantly increased the risk of AE in the second year of the study (Table 5). Further, the presence of respiratory comorbidities in patients with IPF significantly increased risks associated with AE, as well as death (with or without AE), during the entire duration of the study (Table 3 and Table 4). This implies that in patients presenting respiratory comorbidities with IPF, careful monitoring for signs of AE and worsening pulmonary functions is crucial throughout their treatment period. However, the decline in percent predicted FVC and the baseline GAP staging (which includes percent predicted FVC in the staging calculation) were not risk factors for overall survival, possibly because of the poor baseline percent predicted FVC and overall preserved lung function. Results on lung function outcomes, particularly with respect to FVC, were comparable between NICEFIT and INPULSIS serial studies. The mean annual change in FVC (mL) of NICEFIT vs. INPULSIS for the first and second year of observation were −114.3 mL vs. −95 mL and −142.5 mL vs. −150 mL, respectively [13,20]. The NICEFIT treated cohort also had better lung function improvement in comparison to a real-world study of a German cohort, with mean changes in predicted FVC at 6 and 9 months (NICEFIT vs. German cohort: 1.3 ± 10.0% vs. −1.3 ± 7.9% at 6 months and 1.2 ± 13.2% vs. −2.1 ± 9.0% at 9 months) being more stable in the NICEFIT cohort [21]. These differences might be due to dissimilar baseline severities, survivorship bias, smoking status, and government-enabled IPF management programs that facilitate treatment.

With respect to health-related quality of life assessments, the treated group showed relatively better performance in SGRQ (8.4–2.6 points, at week 52–100, Figure 3a), compared to the untreated group (0.2–1.6 points, at week 52–100, Figure S3a) in the present study. However, compared to other studies, the NICEFIT cohort showed poorer performance in SGRQ, with the total SGRQ score being higher (8.4 points, 1 year) than that reported by Richeldi et al. (2.8–4.34 points) [13,22]. A higher number of NICEFIT patients (21.6% in 2 years) faced acute exacerbations compared to INPULSIS and INPULSIS-ON (12% in 4 years) [13,20]. We speculate this to arise from differences in study design (registry vs. interventional), baseline characteristics (e.g., FVC in NICEFIT, 69.7%, and FVC in other studies, 71–80%), and the definition used for classifying acute exacerbation (with respect to the inclusion of COPD-related or other ILD-related acute exacerbations).

Importantly, no new safety concerns were noted with nintedanib treatment in the Taiwanese population. The majority of AEs were mild or moderate in intensity. Compared with the Asian subgroup analysis of the INPULSIS-ON study, the mortality rate in NICEFIT was similar (29.5% in 2 years for NICEFIT, and 28.7% in 5 years for INPULSIS-ON) [23]. This result might be due to baseline parameter variations and the delayed diagnosis of IPF in Taiwan, the analysis of which was beyond the scope of this study.

The small sample size of patients without antifibrotic medication limited statistical evaluation. Further, as NICEFIT is an observational and non-interventional study, there were other challenges to statistical assessments inherent in the study design, such as missing data points for pulmonary and 6MWT function tests owing to the poor health of certain study subjects. Therefore, we applied imputation for missing values in primary and secondary outcomes to improve the statistical fitness of the observed trends.

## 5. Conclusions

NICEFIT is the first long-term, real-world study in Taiwan providing efficacy and safety outcomes for IPF under routine management. Antifibrotic therapy with nintedanib and pirfenidone stabilized lung function parameters in patients with IPF over 2 years of study without increasing mortality, while preserving quality of life. Further, no new safety concerns were indicated for either drug in the Taiwanese population.

## Figures and Tables

**Table 1:** Details of patient demographics and frequently observed comorbidities at the time of enrollment.

Characteristics	Overall	Treated	Untreated	*p*-Value
	N = 101	N = 88	N = 13	
**Age, mean ± SD**	74.6 ± 9.1	74.7 ± 9.0	74.1 ± 10.6	0.822
**Men, n (%)**	84 (83.2)	71 (80.7)	13 (100.0)	0.118
**Weight (kg), mean ± SD**	65.3 ± 12.5	65.7 ± 12.9	62.9 ± 8.8	0.471
**BMI, mean ± SD**	24.6 ± 4.4	24.8 ± 4.6	23.7 ± 3.0	0.448
**Smoking history, n (%)**				
Current	4 (4.0)	2 (2.3)	2 (15.4)	0.086
Former	52 (51.5)	45 (51.1)	7 (53.8)	
Non-smoker	45 (44.6)	41 (46.6)	4 (30.8)	
**Cardio and cerebrovascular comorbidity**				
Arterial hypertension	48 (47.5)	43 (48.9)	5 (38.5)	0.483
Coronary artery disease	19 (18.8)	18 (20.5)	1 (7.7)	0.453
Congestive heart failure	8 (7.9)	7 (8.0)	1 (7.7)	>0.999
Pulmonary hypertension	6 (5.9)	6 (6.8)	0 (0.0)	>0.999
Stroke	5 (5.0)	5 (5.7)	0 (0.0)	>0.999
**Gastrointestinal** **comorbidity**				
GERD	26 (25.7)	23 (26.1)	3 (23.1)	0.356
Gastric ulcer	12 (11.9)	12 (13.6)	0 (0.0)	>0.999
**Metabolic comorbidity**				
DM (type 1 or 2)	22 (21.8)	21 (23.9)	1 (7.7)	0.19
Hyperlipidemia	17 (16.8)	16 (18.2)	1 (7.7)	0.35
**Neoplasm**	7 (6.9)	7 (8.0)	0 (0)	
Colorectal cancer	4 (4.0)	4 (4.5)	0 (0)	>0.999
Prostate cancer	2 (2.0)	2 (2.3)	0 (0)	>0.999
Lung cancer	1 (1.0)	1 (1.1)	0 (0)	>0.999
**Respiratory comorbidity**				
COPD	35 (34.7)	30 (34.1)	5 (38.5)	0.76
Obstructive sleep apnea #	17 (16.8)	16 (18.2)	1 (7.7)	0.35
Emphysema (radiologic)	20 (19.8)	15 (17.0)	5 (38.5)	0.071
Asthma	5 (5.0)	5 (5.7)	0 (0.0)	>0.999

Patient demographics were not significantly different between treated and untreated groups. Comorbidities with an incidence rate > 5% were considered. BMI, body mass index; GERD, gastroesophageal reflux disease; DM, diabetes mellitus; COPD, chronic obstructive pulmonary disease. A *p*-value < 0.05 is significant; # according to results of STOP-Bang scoring model at baseline.

**Table 2:** Baseline readings for primary and secondary outcomes of lung function.

Characteristics	Overall	Treated	Untreated	*p*-Value
Mean ± SD	N = 101	N = 88	N = 13	
**FVC (mL)**	2162.8 ± 645.0	2054.6 ± 592.5	2886.9 ± 513.2	<0.001
**FVC (% predicted)**	73.3 ± 16.6	69.7 ± 14.1	97.8 ± 11.0	<0.001
**DL_CO_ (mL/min/mmHg)**	8.9 ± 4.4	8.8 ± 4.4	9.3 ± 4.4	0.752
**DL_CO_ (% predicted)**	45.2 ± 20.1	42.7 ± 19.7	57.5 ± 18.0	0.025
**TLC (mL)**	3932.0 ± 918.4	3789.0 ± 818.80	5057.5 ± 929.9	<0.001
**TLC (% predicted)**	77.2 ± 15.5	74.3 ± 13.3	100.2 ± 13.0	<0.001
**IC (mL)**	1386.9 ± 493.2	1346.6 ± 478.6	1766.0 ± 518.1	0.07
**IC (% predicted)**	65.6 ± 17.9	62.8 ± 15.6	87.5 ± 21.5	0.052
**SpO_2_ (%)**	95.8 ± 2.3	95.5 ± 2.3	97.5 ± 1.7	0.019
**CAT score**	11.3 ± 7.1	12.0 ± 7.2	6.0 ± 3.3	0.002
**SGRQ score**	33.4 ± 19.8	35.9 ± 19.7	15.8 ± 6.8	<0.001
**6MWT**				
Distance (m)	329.8 ± 96.8	318.5 ± 90.5	419.7 ± 107.1	0.014
Resting SpO_2_ (%)	94.7 ± 3.4	94.4 ± 3.4	96.5 ± 2.6	0.192
Exercise SpO_2_ (%)	90.9 ± 5.72	90.7 ± 5.6	92.5 ± 6.8	0.32
**Obstructive sleep apnea risk ^a^ n (%)**				
Low risk	19 (18.8)	15 (17.0)	4 (30.8)	0.291
Intermediate risk	61 (60.4)	54 (61.4)	7 (53.8)	
High risk	21 (20.8)	19 (21.6)	2 (15.4)	

**^a^** Based on STOP-Bang questionnaire. Primary outcomes included the lung function parameters (i) forced vital capacity (FVC), (ii) diffusion of carbon monoxide in lungs (DL_CO_), (iii) oxygen saturation (SpO_2_), (iv) total lung capacity (TLC), and (v) inspiratory capacity (IC) as measured through spirometry. Secondary outcomes were scored by St. George’s respiratory questionnaire (SGRQ), COPD assessment test (CAT), and 6 min walk test (6MWT). A *p*-value < 0.05 is significant.

**Table 3:** Logistic regression analysis for death between weeks 0–104.

	**n/N (%)**	**Odds Ratio (95% CI)**	***p*-Value**
**All treated patients**	28/88 (31.8)		
**Age (year)**			
<75	13/49 (26.5)		
≥75	15/39 (38.5)	1.891 (0.532, 6.726)	0.325
**Gender**			
Women	2/17 (11.8)		
Men	26/71 (36.6)	9.230 (0.826, 103.114)	0.071
**Smoking**			
Never	14/41 (34.1)		
Current or ex-smoker	14/47 (29.8)	1.049 (0.225, 4.889)	0.951
**BMI**			
<27	23/61 (37.7)		
≥27	5/27 (18.5)	0.364 (0.075, 1.767)	0.21
**GAP stage at baseline**			
Stage I	2/14 (14.3)		
Stage II	9/29 (31.0)	0.936 (0.115, 7.600)	0.951
Stage III	17/44 (38.6)	0.548 (0.061, 4.932)	0.592
**FVC (% pred.) at baseline**			
≥65%	13/52 (25.0)		
<65%	15/35 (42.9)	3.980 (0.994, 15.937)	0.051
**Emphysema at baseline**			
No	24/73 (32.9)		
Yes	4/15 (26.7)	0.411 (0.085, 1.986)	0.269
**Obstructive sleep apnea risk**			
Low risk	4/15 (26.7)		
Intermediate risk	17/54 (31.5)	0.281 (0.031, 2.571)	0.261
High risk	7/19 (36.8)	0.312 (0.021, 4.643)	0.398
**Any comorbidity**			
None	3/10 (30.0)		
Any	25/78 (32.1)	0.140 (0.012, 1.684)	0.121
**Cardiovascular-related comorbidity**			
None	13/37 (35.1)		
Any	15/51 (29.4)	0.823 (0.191, 3.551)	0.794
**Respirator-related comorbidity**			
None	9/36 (25.0)		
Any	19/52 (36.5)	16.286 (1.996, 132.880)	0.009
**Bronchodilator use**			
No	10/39 (25.6)		
Yes	18/49 (36.7)	2.163 (0.564, 8.303)	0.261
**Decline in FVC (% pred.)**			
No	15/56 (26.8)		
Yes	8/23 (34.8)	1.116 (0.245, 5.083)	0.888

BMI, body mass index; CI, confidence interval; FVC, forced vital capacity; GAP, Gender, Age, Physiology; pred, predicted. A *p*-value < 0.05 is significant. Sleep apnea was evaluated according to the STOP-Bang scoring model. Variability in gender, smoke, GAP stage, risk associated with obstructive sleep apnea, or comorbidities were removed from the model where quasi-complete separation of data points were detected.

**Table 4:** Logistic regression analysis for acute exacerbation/death in treated patients between weeks 0–104.

	**n/N (%)**	**Odds Ratio (95% CI)**	***p*-Value**
**All treated patients**	40/88 (45.5)		
**Age (year)**			
<75	18/49 (36.7)		
≥75	22/39 (56.4)	1.882 (0.604, 5.866)	0.275
**Gender**			
Women	4/17 (23.5)		
Men	36/71 (50.7)	6.877 (0.880, 53.745)	0.066
**Smoking**			
Never	21/41 (51.2)		
Current or ex-smoker	19/47 (40.4)	0.530 (0.134, 2.087)	0.364
**BMI**			
<27	33/61 (54.1)		
≥27	7/27 (25.9)	0.393 (0.105, 1.468)	0.165
**GAP stage at baseline**			
Stage I	4/14 (28.6)		
Stage II	11/29 (37.9)	0.713 (0.125, 4.063)	0.703
Stage III	25/44 (56.8)	0.831 (0.135, 5.109)	0.842
**FVC (% pred.) at baseline**			
≥65%	20/52 (38.5)		
<65%	20/35 (57.1)	2.544 (0.740, 8.752)	0.139
**Emphysema at baseline**			
No	34/73 (46.6)		
Yes	6/15 (40.0)	0.470 (0.110, 2.002)	0.307
**Obstructive sleep apnea risk**			
Low risk	5/15 (33.3)		
Intermediate risk	26/54 (48.1)	0.362 (0.048, 2.697)	0.321
High risk	9/19 (47.4)	0.278 (0.023, 3.341)	0.313
**Any comorbidity**			
None	3/10 (30.0)		
Any	37/78 (47.4)	0.737 (0.094, 5.800)	0.772
**Cardiovascular-related comorbidity**			
None	15/37 (40.5)		
Any	25/51 (49.0)	1.577 (0.426, 5.838)	0.495
**Respirator-related comorbidity**			
None	14/36 (38.9)		
Any	26/52 (50.0)	4.848 (1.147, 20.489)	0.032 *
**Bronchodilator use**			
No	18/39 (46.2)		
Yes	22/49 (44.9)	0.896 (0.290, 2.763)	0.848
**Decline in FVC (% pred.)**			
No	22/56 (39.3)		
Yes	13/23 (56.5)	1.203 (0.330, 4.383)	0.779

BMI, body mass index; CI, confidence interval; FVC, forced vital capacity; GAP, Gender, Age, Physiology; pred, predicted. A * *p*-value < 0.05 is significant. Sleep apnea was evaluated according to the STOP-Bang scoring model. Variability in gender, smoke, GAP stage, risk associated with obstructive sleep apnea, or comorbidities were removed from the model where quasi-complete separation of data points were detected.

**Table 5:** Logistic regression analysis for acute exacerbation between weeks 53–104.

	n/N (%)	Odds Ratio (95% CI)	*p*-Value
**All treated patients**	5/88 (5.7)		
**Age (year)**			
<75	4/49 (8.2)		
≥75	1/39 (2.6)	0.235 (0.014, 3.826)	0.309
**BMI**			
<27	2/61 (3.3)		
≥27	3/27 (11.1)	3.978 (0.379, 41.798)	0.25
**FVC (% pred.) at baseline**			
≥65%	3/52 (5.8)		
<65%	2/35 (5.7)	2.276 (0.242, 21.443)	0.472
**Emphysema at baseline**			
No	4/73 (5.5)		
Yes	1/15 (6.7)	1.095 (0.059, 20.410)	0.951
**Cardiovascular-related comorbidity**			
None	1/37 (2.7)		
Any	4/51 (7.8)	6.488 (0.497, 84.764)	0.154
**Respirator-related comorbidity**			
None	2/36 (5.6)		
Any	3/52 (5.8)	0.097 (0.004, 2.415)	0.155
**Bronchodilator use**			
No	1/39 (2.6)		
Yes	4/49 (8.2)	8.860 (0.429, 183.081)	0.158
**Decline in FVC (% pred.)**			
No	2/56 (3.6)		
Yes	3/23 (13.0)	10.887 (1.033, 114.784)	0.047

BMI, body mass index; FVC, forced vital capacity, GAP, Gender-Age-Physiology, pred, predicted. *p*-value < 0.05 is significant.

**Table 6:** Summary of annual change from baseline for secondary outcomes.

Annual Change in SGRQ (Points)		Antifibrotic Drugs N = 88
**Week 52**	Number	57
	Mean ± SD	8.4 ± 16.5
	Median	6.1
	Range	(−30.7, 54.5)
	95% CI	(4.0, 12.8)
**Week 100**	Number	52
	Mean ± SD	2.6 ± 13.4
	Median	0.9
	Range	(−35.6, 32.7)
	95% CI	(−1.1, 6.3)
**Annual change in CAT (points)**		**Antifibrotic drugs** **N = 88**
**Week 52**	Number	56
	Mean ± SD	1.4 ± 7.8
	Median	2.0
	Range	(−16.2, 26.9)
	95% CI	(−0.7, 3.5)
**Week 100**	Number	52
	Mean ± SD	0.7 ± 4.4
	Median	0.5
	Range	(−10.7, 11.2)
	95% CI	(−0.5, 1.9)
**Annual change in 6MWT (meter)**		**Antifibrotic drugs** **N = 88**
**Distance (meter)**		
**Week 52**	Number	24
	Mean ± SD	−7.6 ± 60.4
	Median	0.0
	Range	(−217.9, 92.9)
	95% CI	(−33.1, 17.9)
**Week 100**	Number	15
	Mean ± SD	−20.7 ± 36.6
	Median	-18.8
	Range	(−118.1, 24.5)
	95% CI	(−40.9, −0.4)
**Resting SpO_2_ (%)**		
**Week 52**	Number	24
	Mean ± SD	−0.9 ± 2.6
	Median	−1.2
	Range	(−6.3, 6.0)
	95% CI	(−2.0, 0.3)
**Week 100**	Number	15
	Mean ± SD	0.0 ± 1.5
	Median	0.4
	Range	(−2.4, 2.7)
	95% CI	(−0.9, 0.8)
**Exercise SpO_2_ (%)**		
**Week 52**	Number	24
	Mean ± SD	−1.7 ± 6.2
	Median	−1.9
	Range	(−14.8, 7.3)
	95% CI	(−4.3, 0.9)
**Week 100**	Number	15
	Mean ± SD	−1.1 ± 3.6
	Median	0.0
	Range	(−8.6, 3.5)
	95% CI	(−3.1, 0.9)

SD, standard deviation; CI, confidence interval; 6MWT, six-minute walk test; SpO_2_, oxygen saturation.

## Data Availability

The data presented in this study are available on request from the corresponding author.

## Supplementary Materials

The following supporting information can be downloaded at: https://www.mdpi.com/article/10.3390/biomedicines10102362/s1, Figure S1. Changes in the primary lung function parameters in the treated group. Figure S2. Causes of mortality in the treated group. Figure S3. Secondary outcome trends in the untreated group. Table S1. Summary of antifibrotic treatment. Table S2. Baseline HRCT pattern at the time of enrollment. Table S3. Comorbidity-related medication at the time of enrollment. Table S4. Summary of overall survival in the treated group from 0–104 weeks. Table S5. Safety profile of antifibrotic agents nintedanib and pirfenidone. Table S6. Primary outcome trends in the untreated group. Table S7. Summary of annual change from baseline for secondary outcomes in the untreated group during the study period. Table S8. Summary of overall survival in untreated group. Table S9. Adverse events in the untreated group Supplementary Methods: Data collection procedure.

## Trial Registration NCT03242759

This was a non-interventional multicenter study based on newly collected data on idiopathic pulmonary fibrosis (IPF) patients in clinical practice in Taiwan with a 2-year follow-up period to characterize the IPF population in Taiwan with regard to their clinical course under clinical practice conditions in Taiwan. A total of 101 patients were enrolled across 10 clinical centers in Taiwan between 31 August 2017 to 27 February 2018. In order to eliminate selection bias at the site of treatment, clinics that had access to all treatment options for IPF approved in Taiwan were chosen. Patients were enrolled at the clinics in a consecutive manner to negate selection bias at the patient level.
