# Correction: Cheng et al. NICEFIT—A Prospective, Non-Interventional, and Multicentric Study for the Management of Idiopathic Pulmonary Fibrosis with Antifibrotic Therapy in Taiwan. *Biomedicines* 2022, *10*, 2362

**DOI:** 10.3390/biomedicines13071509

**Published:** 2025-06-20

**Authors:** Shih-Lung Cheng, Chau-Chyun Sheu, Chih-Feng Chian, Jeng-Yuan Hsu, Kuo-Chin Kao, Liang-Wen Hang, Ching-Hsiung Lin, Wen-Feng Fang, Hao-Chien Wang, Diahn-Warng Perng

**Affiliations:** 1 Department of Chemical Engineering and Materials Science, Yuan Ze University, Taoyuan City 320, Taiwan; shihlungcheng@gmail.com; 2 Department of Pulmonary, Far Eastern Memorial Hospital, New Taipei City 220, Taiwan; 3 Division of Pulmonary and Critical Care Medicine, Department of Internal Medicine, Kaohsiung Medical University Hospital, Kaohsiung Medical University, Kaohsiung 807, Taiwan; sheucc@gmail.com; 4 Division of Pulmonary and Critical Care Medicine, Department of Internal Medicine, Tri-Service General Hospital, National Defense Medical Center, Taipei 114, Taiwan; sonice3982@gmail.com; 5 Division of Clinical Research, Taichung Veterans General Hospital, Taichung 407, Taiwan; hsujy@vghtc.gov.tw; 6 Department of Thoracic Medicine, Linkou Chang Gung Memorial Hospital, Taoyuan 333, Taiwan; kck0502@cgmh.org.tw; 7 Department of Pulmonary, China Medical University Hospital, Taichung 404, Taiwan; lungwen.hang@gmail.com; 8 Institute of Genomics and Bioinformatics, National Chung Hsing University, Taichung 402, Taiwan; teddy@cch.org.tw; 9 Ph.D. Program in Translational Medicine, National Chung Hsing University, Taichung 402, Taiwan; 10 Department of Recreation and Holistic Wellness, MingDao University, Changhua 403, Taiwan; 11 Division of Chest Medicine, Department of Internal Medicine, Changhua Christian Hospital, Changhua 403, Taiwan; 12 Department of Internal Medicine, Division of Pulmonary and Critical Care Medicine, Kaohsiung Chang Gung Memorial Hospital, Chang Gung University College of Medicine, Kaohsiung 833, Taiwan; wenfengfang@yahoo.com.tw; 13 Department of Medicine, National Taiwan University Cancer Center, Taipei 100, Taiwan; 14 Department of Chest Medicine, School of Medicine, National Yang-Ming Chiao-Tung University, Taipei Veterans General Hospital, Taipei 112, Taiwan

We would like to identify and amend errors in a previously published paper [1]. The authors state that the scientific conclusions are unaffected. This correction was approved by the Academic Editor. The original publication has also been updated.

## Error in Figure

In the original publication, a component of Figure 2 (Figure 2d) was missing. The data values in the figure are published and cited in the original manuscript text (first paragraph from ‘secondary outcomes’ section). The corrected Figure 2 appears below.

Additionally, incorrect values were included in Figure 3c (the week 52 data value of 6 MWT). Correct values are reported in the original manuscript text (Table 6 and second paragraph from ‘secondary outcomes’ section). The corrected Figure 3 appears below.

## Error in Table

In the original publication, there was a mistake in Tables 3–5 as published, in that 95% CI was incorrectly written as 95% C.I. There was an extra row in Table 3. The corrected Table 3, Table 4 and Table 5 appear below.

## Text Correction

In the Results Section, Section 3.2.1., Figure 2a was mis-cited in the second sentence of paragraphs 1 and 2 and they should be Figure S1. The corrected sentences appear below.

The mean ± SD of absolute annual change from baseline in FVC was −114.3 ± 441.5 mL at week 52 and −142.5 ± 610.8 mL at week 100 (Figure S1).

There was no significant change from baseline (mean ± SD) for absolute DL_CO_, which ranged from −2.6 to 0.1 mL/min/mmHg (Figure S1) during the 2-year follow-up.

In the Discussion section, there was an error in the original publication. The “mean ± 1.7%” is updated to “±1.7%” in the first paragraph of the discussion for consistency with the results. The corrected sentence appears below.

Despite significantly compromised baseline functionality, antifibrotic therapy with nintedanib or pirfenidone limited further deteriorations of respiratory functions, especially with respect to annual changes from baseline in percent predicted FVC (±1.7%), without adversely affecting the quality of life.

## Modifications in Author Contributions

The corrected Author Contributions appears below.

Conceptualization, S.-L.C., H.-C.W. and D.-W.P.; validation, H.-C.W. and D.-W.P.; investigation, S.-L.C., C.-C.S., C.-F.C., J.-Y.H., K.-C.K., L.-W.H., C.-H.L., W.-F.F., H.-C.W. and D.-W.P.; data curation, S.-L.C., C.-C.S., C.-F.C., J.-Y.H., K.-C.K., L.-W.H., C.-H.L., W.-F.F., H.-C.W. and D.-W.P.; writing—review and editing, S.-L.C.; visualization, S.-L.C., H.-C.W. and D.-W.P. All authors have read and agreed to the published version of the manuscript.

## Figures and Tables

**Figure 2:**
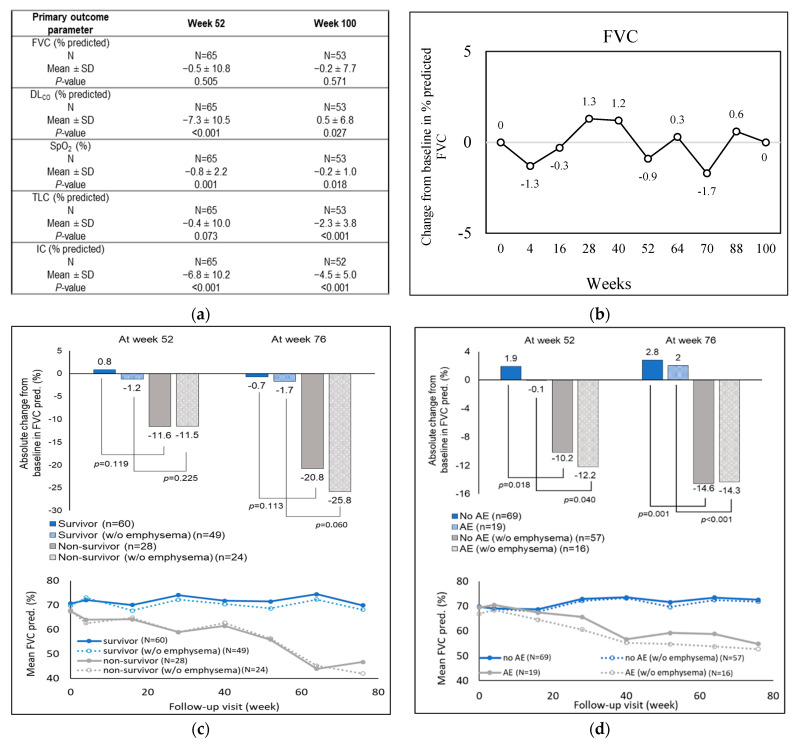
Changes in baseline of primary outcome parameters across the study period in the treated group. (**a**) Annual changes from baseline for the primary lung function parameters, (i) FVC, (ii) DL_CO_, (iii) SpO_2_, (iv) TLC, and (v) IC, as measured through spirometry. (**b**) Changes in percent predicted FVC from baseline. (**c**) Predicted mean and absolute FVC changes in survivors and non-survivors, treated with antifibrotic drugs, and (**d**) predicted mean and absolute FVC changes in treated patients experiencing versus not experiencing at least one acute exacerbation, stratified by emphysema status. AE, acute exacerbation; DL_CO_, diffusion of carbon monoxide in lungs; FVC, forced vital capacity; IC, inspiratory capacity; SpO_2_, oxygen saturation; pred, predicted; TLC, total lung capacity.

**Figure 3:**
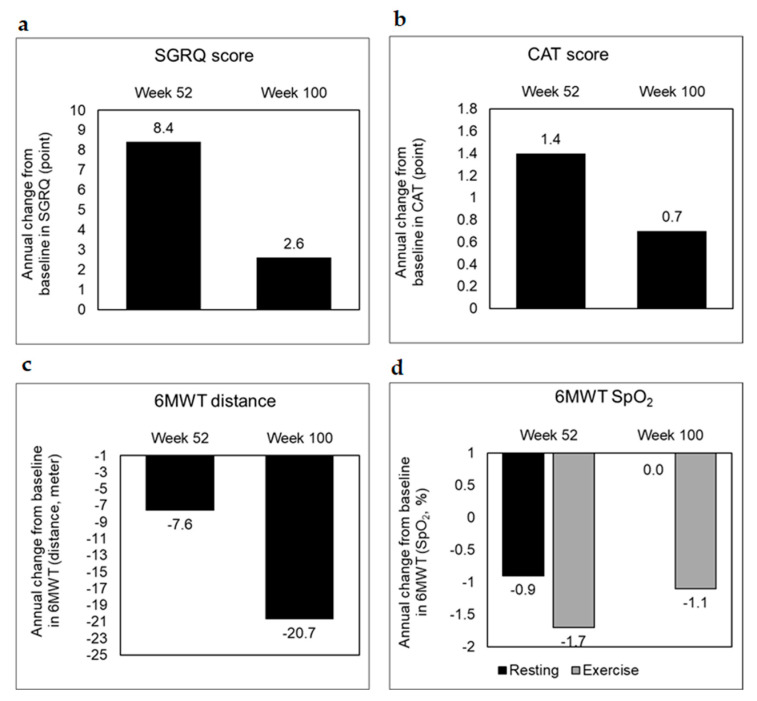
Secondary outcome trends in the treated group. Secondary outcomes with respect to (**a**) SGRQ, (**b**) CAT, and (**c**,**d**) 6MWT were scored as annual changes from the baseline at the end of weeks 52 and 100 to assess health-related quality of life, airway obstruction and exercise-related pulmonary function, respectively. SGRQ, St. George’s respiratory questionnaire; CAT, chronic obstructive pulmonary disease assessment test; 6MWT, 6 min walk test.

**Table 3:** Logistic regression analysis for death between weeks 0–104.

	n/N (%)	Odds Ratio (95% CI)	*p*-Value
**All treated patients**	28/88 (31.8)		
**Age (year)**			
<75	13/49 (26.5)		
≥75	15/39 (38.5)	1.891 (0.532, 6.726)	0.325
**Gender**			
Women	2/17 (11.8)		
Men	26/71 (36.6)	9.230 (0.826, 103.114)	0.071
**Smoking**			
Never	14/41 (34.1)		
Current or ex-smoker	14/47 (29.8)	1.049 (0.225, 4.889)	0.951
**BMI**			
<27	23/61 (37.7)		
≥27	5/27 (18.5)	0.364 (0.075, 1.767)	0.21
**GAP stage at baseline**			
Stage I	2/14 (14.3)		
Stage II	9/29 (31.0)	0.936 (0.115, 7.600)	0.951
Stage III	17/44 (38.6)	0.548 (0.061, 4.932)	0.592
**FVC (% pred.) at baseline**			
≥65%	13/52 (25.0)		
<65%	15/35 (42.9)	3.980 (0.994, 15.937)	0.051
**Emphysema at baseline**			
No	24/73 (32.9)		
Yes	4/15 (26.7)	0.411 (0.085, 1.986)	0.269
**Obstructive sleep apnea risk**			
Low risk	4/15 (26.7)		
Intermediate risk	17/54 (31.5)	0.281 (0.031, 2.571)	0.261
High risk	7/19 (36.8)	0.312 (0.021, 4.643)	0.398
**Any comorbidity**			
None	3/10 (30.0)		
Any	25/78 (32.1)	0.140 (0.012, 1.684)	0.121
**Cardiovascular-related comorbidity**			
None	13/37 (35.1)		
Any	15/51 (29.4)	0.823 (0.191, 3.551)	0.794
**Respirator-related comorbidity**			
None	9/36 (25.0)		
Any	19/52 (36.5)	16.286 (1.996, 132.880)	0.009
**Bronchodilator use**			
No	10/39 (25.6)		
Yes	18/49 (36.7)	2.163 (0.564, 8.303)	0.261
**Decline in FVC (% pred.)**			
No	15/56 (26.8)		
Yes	8/23 (34.8)	1.116 (0.245, 5.083)	0.888

BMI, body mass index; CI, confidence interval; FVC, forced vital capacity; GAP, Gender, Age, Physiology; pred, predicted. A *p*-value < 0.05 is significant. Sleep apnea was evaluated according to the STOP-Bang scoring model. Variability in gender, smoke, GAP stage, risk associated with obstructive sleep apnea, or comorbidities were removed from the model where quasi-complete separation of data points were detected.

**Table 4:** Logistic regression analysis for acute exacerbation/death in treated patients between weeks 0–104.

	n/N (%)	Odds Ratio (95% CI)	*p*-Value
**All treated patients**	40/88 (45.5)		
**Age (year)**			
<75	18/49 (36.7)		
≥75	22/39 (56.4)	1.882 (0.604, 5.866)	0.275
**Gender**			
Women	4/17 (23.5)		
Men	36/71 (50.7)	6.877 (0.880, 53.745)	0.066
**Smoking**			
Never	21/41 (51.2)		
Current or ex-smoker	19/47 (40.4)	0.530 (0.134, 2.087)	0.364
**BMI**			
<27	33/61 (54.1)		
≥27	7/27 (25.9)	0.393 (0.105, 1.468)	0.165
**GAP stage at baseline**			
Stage I	4/14 (28.6)		
Stage II	11/29 (37.9)	0.713 (0.125, 4.063)	0.703
Stage III	25/44 (56.8)	0.831 (0.135, 5.109)	0.842
**FVC (% pred.) at baseline**			
≥65%	20/52 (38.5)		
<65%	20/35 (57.1)	2.544 (0.740, 8.752)	0.139
**Emphysema at baseline**			
No	34/73 (46.6)		
Yes	6/15 (40.0)	0.470 (0.110, 2.002)	0.307
**Obstructive sleep apnea risk**			
Low risk	5/15 (33.3)		
Intermediate risk	26/54 (48.1)	0.362 (0.048, 2.697)	0.321
High risk	9/19 (47.4)	0.278 (0.023, 3.341)	0.313
**Any comorbidity**			
None	3/10 (30.0)		
Any	37/78 (47.4)	0.737 (0.094, 5.800)	0.772
**Cardiovascular-related comorbidity**			
None	15/37 (40.5)		
Any	25/51 (49.0)	1.577 (0.426, 5.838)	0.495
**Respirator-related comorbidity**			
None	14/36 (38.9)		
Any	26/52 (50.0)	4.848 (1.147, 20.489)	0.032 *
**Bronchodilator use**			
No	18/39 (46.2)		
Yes	22/49 (44.9)	0.896 (0.290, 2.763)	0.848
**Decline in FVC (% pred.)**			
No	22/56 (39.3)		
Yes	13/23 (56.5)	1.203 (0.330, 4.383)	0.779

BMI, body mass index; CI, confidence interval; FVC, forced vital capacity; GAP, Gender, Age, Physiology; pred, predicted. A * *p*-value < 0.05 is significant. Sleep apnea was evaluated according to the STOP-Bang scoring model. Variability in gender, smoke, GAP stage, risk associated with obstructive sleep apnea, or comorbidities were removed from the model where quasi-complete separation of data points were detected.

**Table 5:** Logistic regression analysis for acute exacerbation between weeks 53–104.

	n/N (%)	Odds Ratio (95% CI)	*p*-Value
**All treated patients**	5/88 (5.7)		
**Age (year)**			
<75	4/49 (8.2)		
≥75	1/39 (2.6)	0.235 (0.014, 3.826)	0.309
**BMI**			
<27	2/61 (3.3)		
≥27	3/27 (11.1)	3.978 (0.379, 41.798)	0.25
**FVC (% pred.) at baseline**			
≥65%	3/52 (5.8)		
<65%	2/35 (5.7)	2.276 (0.242, 21.443)	0.472
**Emphysema at baseline**			
No	4/73 (5.5)		
Yes	1/15 (6.7)	1.095 (0.059, 20.410)	0.951
**Cardiovascular-related comorbidity**			
None	1/37 (2.7)		
Any	4/51 (7.8)	6.488 (0.497, 84.764)	0.154
**Respirator-related comorbidity**			
None	2/36 (5.6)		
Any	3/52 (5.8)	0.097 (0.004, 2.415)	0.155
**Bronchodilator use**			
No	1/39 (2.6)		
Yes	4/49 (8.2)	8.860 (0.429, 183.081)	0.158
**Decline in FVC (% pred.)**			
No	2/56 (3.6)		
Yes	3/23 (13.0)	10.887 (1.033, 114.784)	0.047

BMI, body mass index; FVC, forced vital capacity, GAP, Gender-Age-Physiology, pred, predicted. *p*-value < 0.05 is significant.
