# Supplementary material for: NICEFIT—A Prospective, Non-Interventional, and Multicentric Study for the Management of Idiopathic Pulmonary Fibrosis with Antifibrotic Therapy in Taiwan
Source: Biomedicines. 2022 Sep 22;10(10):2362. doi: 10.3390/biomedicines10102362 (PMC9598748; doi:10.3390/biomedicines10102362)
Supplement: Supplementary file 1 [file biomedicines-10-02362-s001.zip › biomedicines-1792413-supplementary.pdf]

Supplementary figures and tables

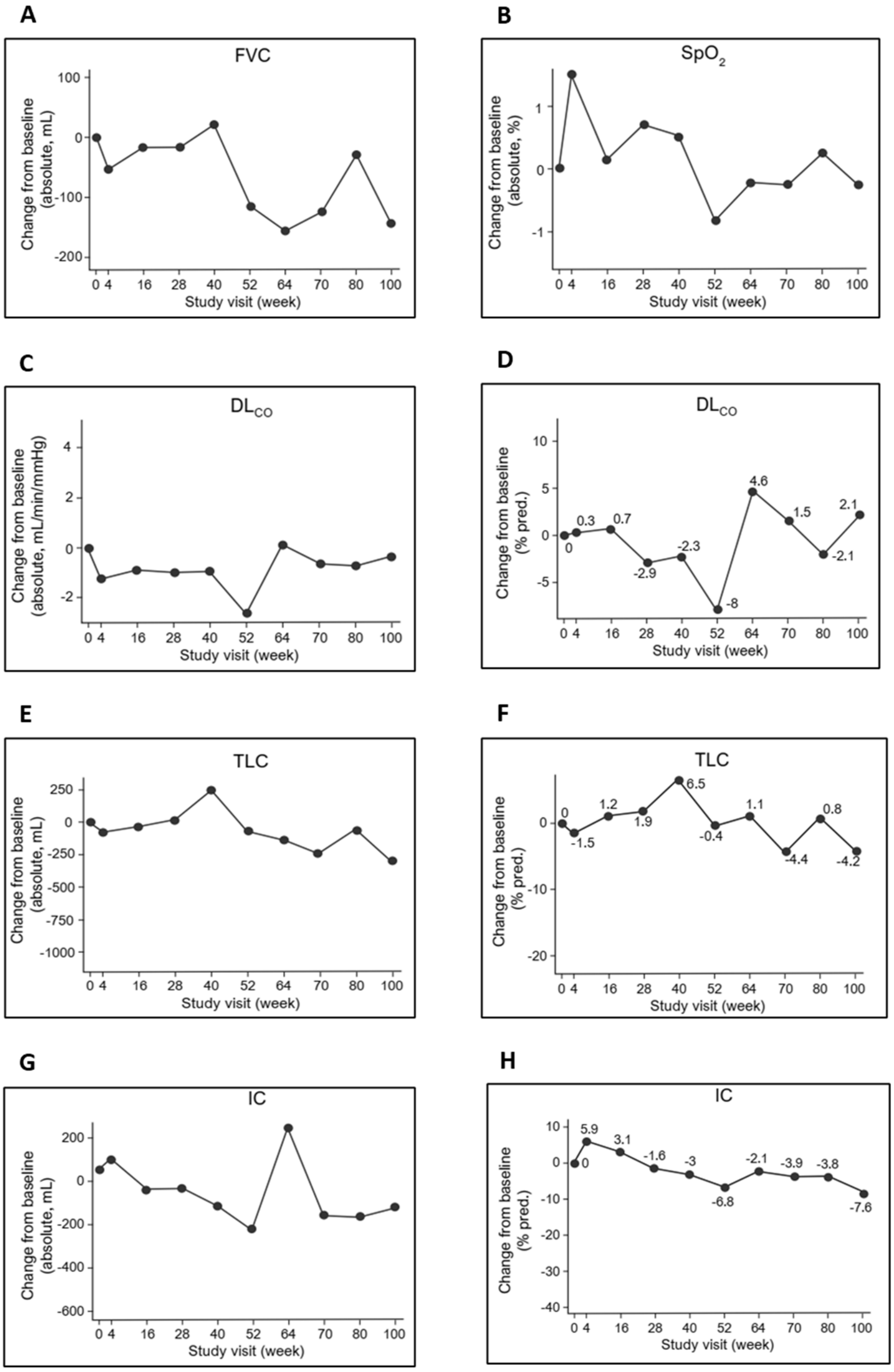

Figure S1. Changes in the primary lung function parameters in the treated group. Changes in baseline of the primary outcome parameters (A) forced vital capacity (FVC), (B) oxygen saturation

(SpO_2), (C, D) diffusion of carbon monoxide in lungs (DL_{CO}), (E, F) total lung capacity (TLC), and (G, H) inspiratory capacity (IC) as measured through spirometry.

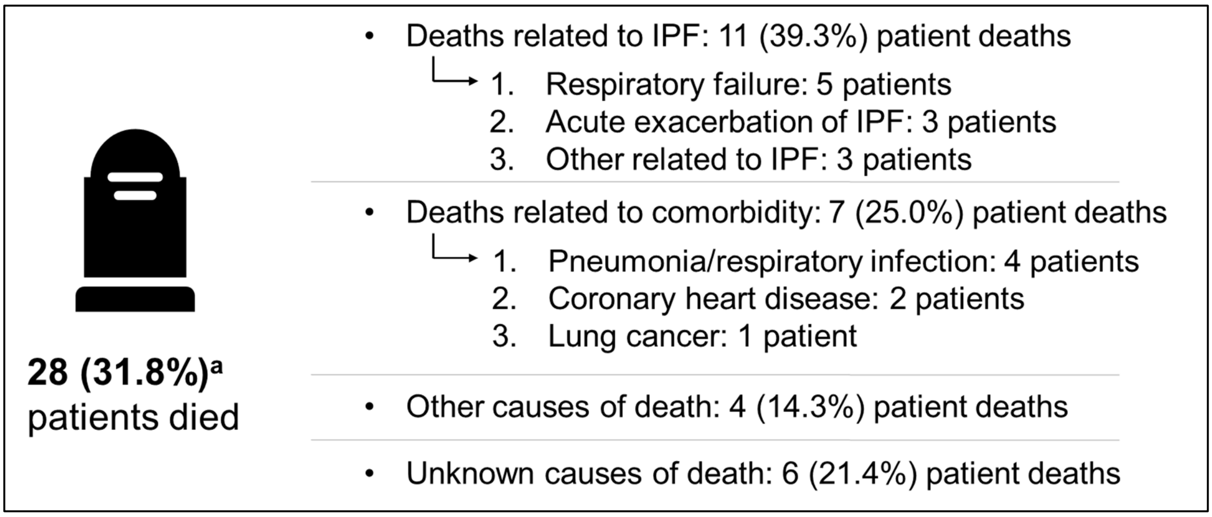

Figure S2. Causes of mortality in the treated group. [a]Out of 88 treated patients 28 died. IPF, idiopathic pulmonary fibrosis

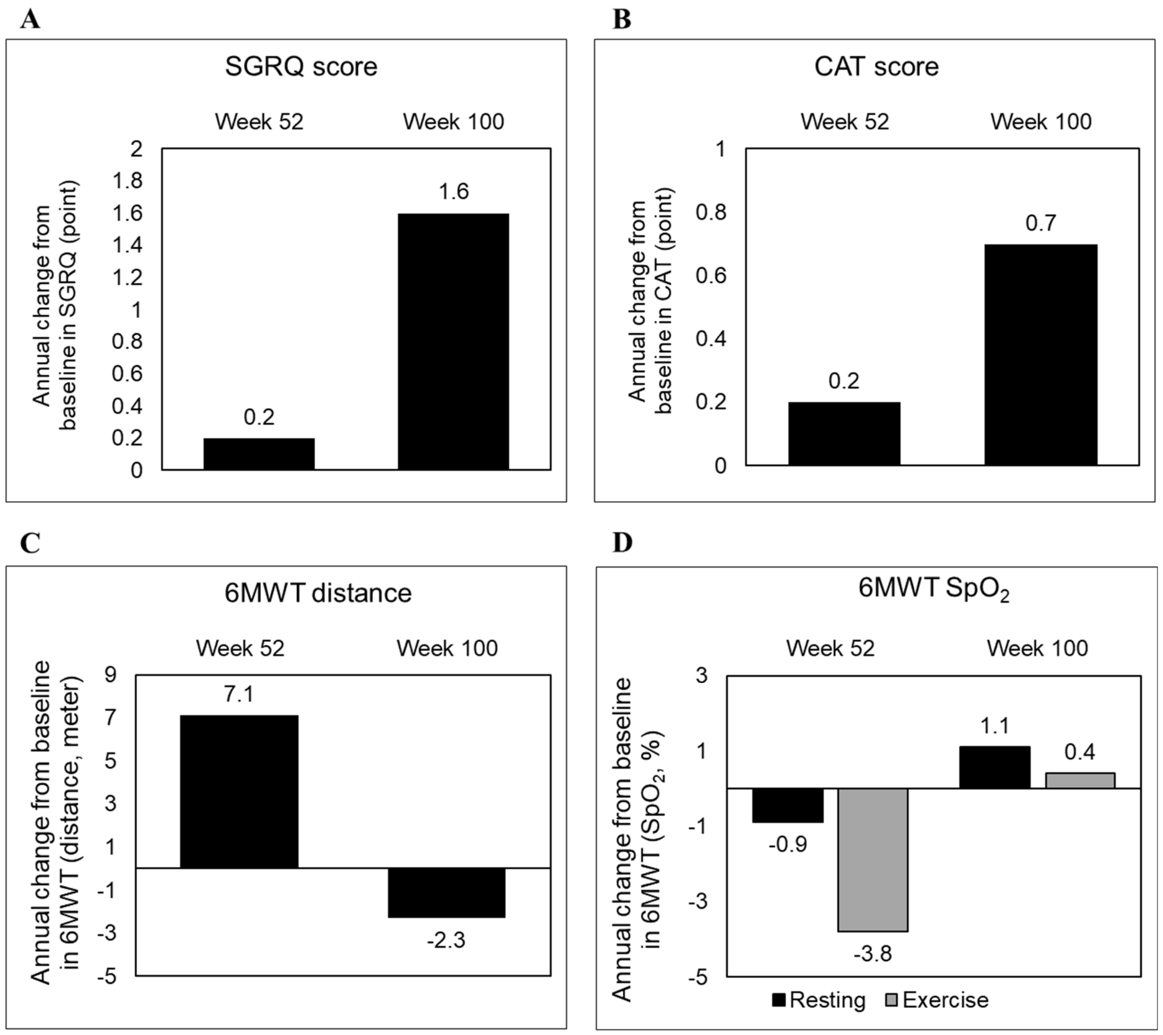

Figure S3. Secondary outcome trends in the treated group. Secondary outcomes with respect to (**A**) SGRQ. (**B**) CAT, and (**C, D**) 6MWT were scored as annual changes from the baseline at the end of weeks 52 and 100, to assess health-related quality of life, airway obstruction, and exercise-related pulmonary function, respectively.

Table S1. Summary of antifibrotic treatment.

Characteristics	Treated patients<br>N = 88
**Anti-Fibrotic Drugs, n (%)**	
Nintedanib	73 (83.0)
Pirfenidone	5 (5.7)
Both nintedanib and pirfenidone	
Switch	9 (10.2)
Contemporary use	1 (1.1)
**Reason of switch, n (%)**	
Adverse event(s)	6 (60.0)
Disease progression	2 (20.0)
Withdraw	1 (10.0)
**Average dose intensity of Nintedanib, %**	88.2
**Initial dose of Nintedanib, n (%)**	
150 mg BID	73 (88.0)
150 mg QD	9 (10.8)
150 mg QOD	1 (1.2)
**Average dose intensity of Pirfenidone, %**	46.6
**Initial dose of Pirfenidone, n (%)**	
267 mg TID, week 1	15 (17.0)
534 mg TID, week 2	
801 mg TID, week 3 onwards	

Contemporary refers to concomitant medication with nintedanib and pirfenidone. QD, once daily; QOD, once every alternate day; BID, twice daily; TID, thrice daily.

Table S2. Baseline HRCT pattern at the time of enrolment.

Characteristics	Overall N = 101	Treated N = 88	Untreated N = 13	P-value
**HRCT pattern, n (%)**				
Definite UIP	72 (71.3)	63 (71.6)	9 (69.2)	0.698
Possible UIP	23 (22.8)	20 (22.7)	3 (23.1)	
Inconsistent	3 (3.0)	3 (3.4)	0 (0.0)	
**Of patients with definite or possible UIP, n (%)**				
Subpleural, basal predominance	85 (89.5)	73 (88.0)	12 (100.0)	0.352
Honeycombing with or without traction	82 (86.3)	72 (86.7)	10 (83.3)	0.667
Reticular abnormality	68 (71.6)	60 (72.3)	8 (66.7)	0.736
Absence of features listed as inconsistent with UIP pattern	39 (41.1)	33 (39.8)	6 (50.0)	0.542

HRCT, high resolution computerized tomography. P-value <0.05 is significant.

Table S3. Comorbidity-related medication at the time of enrolment.

Characteristics<br>N (%)	Overall<br>N = 101	Treated<br>N = 88	Untreated<br>N = 13	P-value
**Respiratory**	66 (65.3)	60 (68.2)	6 (46.2)	0.132
**Hypertension**	21 (20.8)	21 (23.9)	0 (0.0)	0.065
**CVD/cerebral artery occlusion**	18 (17.8)	17 (19.3)	1 (7.7)	0.454
**DM**	14 (13.9)	14 (15.9)	0 (0.0)	0.205
**Hyperlipidemia**	9 (8.9)	9 (10.2)	0 (0.0)	0.600

CVD, cardiovascular disease; DM, diabetes mellitus. P-value <0.05 is significant.

Table S4. Summary of overall survival in the treated group from 0-104 weeks.

	**Anti-fibrotic drugs**<br>**N = 88**
**Death, n (%)**	28 (31.8)
**Survival time (days)**	
**Median (95% CI)**	NA (708.0, NA)
**Follow-up period for overall survival (days)**	
**Number**	88
**Mean ± SD**	541.1 ± 244.82
**Median**	686.0
**Range**	(14.0, 899.0)
**95% CI**	(489.2, 593.0)

SD, standard deviation; CI, confidence interval.

Table S5. Safety profile of antifibrotic agents nintedanib and pirfenidone.

Adverse events, n (%)	Anti-fibrotic drugs (N = 88)
**Patients with AEs by severity**	
Mild	39 (44.3)
Moderate	15 (17.0)
Severe	33 (37.5)
Unknown	1 (1.1)
**Gastrointestinal disorders**	**35 (39.8)**
Diarrhoea	29 (33.0)
Gastric ulcer	2 (2.3)
Nausea	2 (2.3)
Abdominal pain upper	1 (1.1)
Dry mouth	1 (1.1)
Gastritis	1 (1.1)
Upper gastrointestinal haemorrhage	1 (1.1)
Vomiting	1 (1.1)
**Respiratory, thoracic and mediastinal**	**16 (18.2)**
**disorders**	9 (10.2)
Idiopathic pulmonary fibrosis	4 (4.5)
Respiratory failure	1 (1.1)
Chronic obstructive pulmonary disease	1 (1.1)
Dysphonia	1 (1.1)
Dyspnoea	1 (1.1)
Epiglottic cyst	1 (1.1)
Pneumonia aspiration	1 (1.1)
Pneumonitis	0 (0.0)
Pulmonary mass	
**Hepatobiliary disorders**	**5 (5.7)**
Hepatic function abnormal	3 (3.4)
Hepatitis	2 (2.3)
Hepatic cirrhosis	1 (1.1)

Overall drug-related adverse events in the treated group.

Table S6. Primary outcome trends in the untreated group.

Primary outcome parameters	Week 52	Week 100
**FVC (mL)**	52.0 ± 157.86	−210.0 ± 231.62
**FVC (% predicted.)**	4.1 ± 7.73	−2.5 ± 4.52
**DL_{CO} (mL/min/mmHg)**	−0.1 ± 1.93	−1.2 ± 2.49
**DL_{CO} (% predicted.)**	−2.8 ± 8.27	−2.6 ± 6.26
**SpO_2 (%)**	−0.8 ± 0.92	−0.6 ± 0.84
**TLC (mL)**	−240.0 ± 778.72	−340.0 ± 757.17
**TLC (% predicted.)**	−1.4 ± 12.18	−3.4 ± 6.89
**IC (mL)**	−70.0 ± 14.14	−145.0 ± 106.07
**IC (% predicted.)**	−6.1 ± 1.73	−6.0 ± 2.40

Annual changes from baseline for the primary lung function parameters (i) forced vital capacity (FVC), (ii) diffusion of carbon monoxide in lungs (DL_{CO}), (iii) oxygen saturation (SpO_2), (iv) total lung capacity (TLC), and (v) inspiratory capacity (IC) as measured through spirometry.

Table S7. Summary of annual change from baseline for secondary outcomes in the untreated group during the study period

**Annual change in SGRQ (points)**		**Non-anti-fibrotic drugs<br>N = 13**
**Week 52**	Number	8
	Mean ± SD	0.2 ± 8.12
	Median	1.9
	Range	(-13.9, 12.8)
	95% CI	(-6.6, 7.0)
**Week 100**	Number	6
	Mean ± SD	1.6 ± 6.17
	Median	1.6
	Range	(-7.0, 10.0)
	95% CI	(-4.8, 8.1)
**Annual change in CAT (points)**		**Non-anti-fibrotic drugs<br>N = 13**
**Week 52**	Number	8
	Mean ± SD	0.2 ± 1.96
	Median	0.5
	Range	(-3.3, 2.8)
	95% CI	(-1.4, 1.8)
**Week 100**	Number	6
	Mean ± SD	0.7 ± 2.13
	Median	0.0
	Range	(-2.0, 4.1)
	95% CI	(-1.5, 2.9)
**Annual change in 6MWT (meter)**		**Non-anti-fibrotic drugs<br>N = 13**
**Distance (meter)**		
**Week 52**	Number	3
	Mean ± SD	7.1 ± 31.53
	Median	0.0
	Range	(-20.2, 41.6)
	95% CI	(-71.2, 85.5)
**Week 100**	Number	2
	Mean ± SD	-2.3 ± 20.51
	Median	-2.3
	Range	(-16.8, 12.2)

	95% CI	(-186.6, 181.9)
**Resting SpO_2 (%)**		
**Week 52**	Number	3
	Mean ± SD	-0.9 ± 0.87
	Median	-1.0
	Range	(-1.7, 0.0)
	95% CI	(-3.1, 1.3)
**Week 100**	Number	2
	Mean ± SD	1.1 ± 1.02
	Median	1.1
	Range	(0.4, 1.8)
	95% CI	(-8.1, 10.3)
**Exercise SpO_2 (%)**		
**Week 52**	Number	3
	Mean ± SD	-3.8 ± 4.13
	Median	-3.1
	Range	(-8.2, 0.0)
	95% CI	(-14.0, 6.5)
**Week 100**	Number	2
	Mean ± SD	0.4 ± 0.54
	Median	0.4
	Range	(0.0, 0.8)
	95% CI	(-4.5, 5.3)

SD, standard deviation; CI, confidence interval; 6MWT, Six-Minute Walk Test; SpO_2, oxygen saturation.

Table S8. Summary of overall survival in untreated group

	Non-anti-fibrotic drugs N = 13
**Death, n (%)**	1 (7.7)
**Survival time (days)**	
Median (95% CI)	NA (596.0, NA)
**Follow-up period for overall survival (days)**	
Number	12
Mean ± SD	515.4 ± 228.70
Median	641.0
Range	(111.0, 721.0)
95% CI	(370.1, 660.7)

CI, confidence interval; SD, standard deviation; NA, not available.

Table S9. Adverse events in the untreated group

Adverse events	Untreated N = 13, n (%)
**Cardiac disorders**	2 (15.4)
Cardiac arrest	1 (7.7)
Acute myocardial infarction	1 (7.7)
**Respiratory, thoracic and mediastinal disorders**	1 (7.7)
Pulmonary mass	1 (7.7)
**Investigations**	1 (7.7)
Alanine aminotransferase abnormal	1 (7.7)

CI, confidence interval; SD, standard deviation; NA, not available.

Supplementary Methods

Data collection procedure

Data for baseline and follow-up visits, medical history of IPF and co-morbidities: Data were collected for age, gender, race, education, environmental or occupational exposures, height, weight, body mass index (BMI), and smoking history (current smokers, ex-smokers, or non-smokers). Data on medical history of IPF included the date of IPF diagnosis, details diagnostic procedure (e.g., HRCT, surgical lung biopsy, multidisciplinary team [MDT] diagnosis) and the result of diagnosis (e.g., UIP pattern). Past or active status of relevant comorbidities, such as, cardio and cerebrovascular (e.g., deep vein thrombosis, pulmonary embolism, coronary artery disease), respiratory (e.g., COPD, obstructive sleep apnea according to STOP-Bang scoring at baseline), renal or hepatic (hepatic cirrhosis), gastrointestinal (e.g., gastroesophageal reflux disease or GERD), metabolic (e.g., Type I/ Type II diabetes mellitus and hyperlipidemia), and neoplasms, were recorded. Comorbidities related to concomitant medication within 6 months from date of enrolment were also recorded to show dates of initiation and competition of medication, and dosage. IPF-related clinical symptoms such as cough, dyspnea, fatigue, weight loss, clubbed fingers, respiratory aid, muscle or joint pain, chest pain, and anxiety, were also recorded.

Serological and biomarker tests: Results for serological and biomarker tests available on medical charts were collected as potential links to the prognosis of IPF in patients. Serological test data was collected for weeks 15, 62, 76, and 100, whereas biomarkers were assessed for weeks 52 and 100. The serological markers scored were Hematocrit (Hct), hemoglobin (Hb), platelets, RBC, WBC, neutrophils, lymphocytes, monocytes, eosinophils, basophils, C-reactive protein (CRP), creatine kinase (CK), rheumatoid factor, antinuclear antibody, anti-Jo1 antibody, Sjogren's SS-A or SS-B antibody, seleroderma-70 antibody, cytoplasmic anti-neutrophil cytoplasmic antibody (c-ANCA), and perinuclear anti-neutrophil cytoplasmic antibody (p-ANCA). The biomarkers scored were VEGF, fibroblast growth factor , and PDGF.

Gene polymorphism: Data on gene polymorphisms implicated in IPF (such as MUC 5B) were collected at baseline if they were available from patient medical records.
